# Supplementary material for: Patient-Reported Experience Measures for Colonoscopy: A Systematic Review and Meta-Ethnography
Source: Diagnostics (Basel). 2022 Jan 19;12(2):242. doi: 10.3390/diagnostics12020242 (PMC8871001; doi:10.3390/diagnostics12020242)
Supplement: Supplementary file 1 [file diagnostics-12-00242-s001.zip › Table_S1_Concepts_3order_LoA_211226.pdf]

diagnostics

MDPI

Table S1. Synthesis, including concepts, third-order constructs, and Line of Argument.

Concept	Third-order constructs	Line of Argument
**Health motivation**. What motivated the adult patients to go through with the colonoscopy.	Adult patients who have undergone a colonoscopy, whether they have symptoms or not, have a desire to be healthy and to determine their current state of bowel health.	Experienced good health is a desirable state of being.
**Discomfort**. Experienced discomfort before during and after the procedure.	Patients make logistical plans prior to a colonoscopy, they hesitate to undergo the bowel preparation and, if current, they experience concerns regarding if the procedure exacerbate their pre-existing conditions.	Colonoscopy is an inconvenient procedure for the patients which can be alleviated by the support of healthcare professionals.
	A colonoscopy places the patients in an awkward and embarrassing situation due to the procedure's sensitive nature.	
	The patients experience of discomfort and/or pain during the procedure differ.	
	After a colonoscopy procedure the patients' needs to recover, both mentally and physically.	
	Healthcare professionals can guide patients to tolerate experienced discomfort and/or pain during the colonoscopy and for some patients, sedation can moderate and ease these experiences.	
**Information**. How patients talk with others and read about the colonoscopy before the procedure. Information given to the patients by the healthcare professionals during the colonoscopy as well as the results after the procedure.	Patients have different conditions to assimilate information and to communicate about the colonoscopy prior to the procedure.	Beneficial patient experiences are created by sharing of information.
	Provided it's in accordance with the patient's preferences, healthcare professionals sharing of verbal and visual information during the colonoscopy make the patients feel involved and treated as a partner.	
	The patients greatly value a result that is disclosing information about their bowel condition before discharge and, if not given this, they experience frustration.	
**A caring relationship**. Interaction during the colonoscopy between patients and healthcare professionals.	A caring relationship is built on trust where individual needs are being met and when a respectful interaction is present between the patients and the healthcare professionals.	Mutual respect and trust are the foundation to a caring relationship between the patient and healthcare professionals.
	When a caring relationship is created, the patient's voluntary gives away control, if needed, to the healthcare professionals during the colonoscopy.	

Table S1. Continued.

Concept	Third-order constructs	Line of Argument
**Understanding**. What the patients think will happen and how they believe they will experience to undergo a colonoscopy. What happened and how the patients actually experienced to undergo a colonoscopy	Patients face the unknown by anticipating their experiences of an impending colonoscopy and their understanding for the procedure influence their perception of it.	The patients' understanding prior to the colonoscopy is re-evaluated by them after the experienced procedure
	Negative emotions and expectations are common before the procedure due to individual references and social context.	
	Going through a colonoscopy entails the understanding of that believes concerning the procedure do not replace an actual experience of it.	
	Negative experiences of a colonoscopy are associated with the patient's willingness to repeat the procedure.	
