# Supplementary material for: Patient-Reported Experience Measures for Colonoscopy: A Systematic Review and Meta-Ethnography
Source: Diagnostics (Basel). 2022 Jan 19;12(2):242. doi: 10.3390/diagnostics12020242 (PMC8871001; doi:10.3390/diagnostics12020242)
Supplement: Supplementary file 1 [file diagnostics-12-00242-s001.zip › Table_S2_Contribution_to_Synthesis_211226.pdf]

Table S2. Overview of how each included study have contributed to the five main concepts and the Line of Argument.

Studies	Health motivation n=10	Discomfort n=13			Information n=11			Care relationship n=11			Understanding n=9		
	Overall	Before	During	After	Before	During	After	Before	During	After	Before	During	After
**Hafeez et al. [33]**			√			√	√						
**Kimura et al. [34]**	√	√							√		√		
**Kirkegaard et al. [35]**	√	√	√			√	√		√				
**Mikocka-Walus et al. [36]**	√	√			√		√		√		√		√
**Neilson et al. [37]**		√	√		√		√		√		√		
**Restall et al. [38]**	√	√			√		√		√		√		√
**Rollbusch et al. [39]**	√		√		√		√		√		√		√
**Rosvall et al. [32]**	√	√	√	√	√	√	√		√		√		√
**Shamim et al. [40]**	√	√	√		√		√		√		√		√
**Sultan et al. [41]**	√	√									√		
**Thygesen et al. [42]**	√	√	√				√		√				
**von Wagner et al. [43]**	√		√			√	√		√				
**Wangmar et al. [44]**		√	√	√	√	√			√		√		√
